# Invasive sarcomatoid mesothelioma resulting in spinal cord compression: case report

**DOI:** 10.1259/bjrcr.20170068

**Published:** 2017-11-10

**Authors:** Matthew Farthing, Thurkaa Shanmugalingam, Elizabeth Alice Dean, Dakshinamoorthy Muthukumar

**Affiliations:** Oncology, Colchester Hospital University Foundation Trust, Cancer Services, Colchester, Essex, UK

## Abstract

Mesothelioma is more likely to metastasize by local invasion, and metastases to the nervous system are rare. There are currently 10 reported cases of spinal cord compression as a result of mesothelioma. We report a 74-year-old patient with sarcomatoid mesothelioma that spreads across the dura into the spinal cord at T4/T5 level. This case report illustrates an unusual presentation of spinal cord compression by mesothelioma. It details the presenting symptoms, examinations and management of the patient and provides an overview of other potential metastatic sites of mesothelioma.

## Patient history

A 74-year-old man was admitted with ongoing back pain that had occurred 2 months previously. He reported the pain to be of maximum intensity in the midthoracic region, which radiated down the lumbar spine through to the limbs. His mobility worsened over this period and in addition to the back pain he presented with “off legs.” This was also associated with faecal and urinary incontinence. He had a past history of significant occupational exposure to asbestos during his work as a carpenter.

His past medical history includes locally advanced prostate cancer, diagnosed in 2006 and managed with a radical prostatectomy and adjuvant radiotherapy. The prostate-specific antigen levels remained at a plateau on the current admission. In 2013, on routine CT imaging an abnormality revealed lobulated pleural thickening in the left lower lobe. Following one failed and then one successful biopsy, the patient was diagnosed with sarcomatoid mesothelioma. He underwent six cycles of chemotherapy (cisplatin/pemetrexed) and the disease remained fairly stable. In mid-2016 there was further disease progression and he was offered further six cycles of cisplatin/pemetrexed, which again stabilized the disease.

## Clinical findings

He was cachectic, and physical examination revealed that the lower limbs were weak—power 2/5 in hip flexors and 4/5 in knee extension and flexion and ankle plantar flexion and dorsiflexion. No loss of sensation and hyper-reflexia in all lower deep tendon reflexes was reported.

## Investigation and image findings

**Figure 1.**
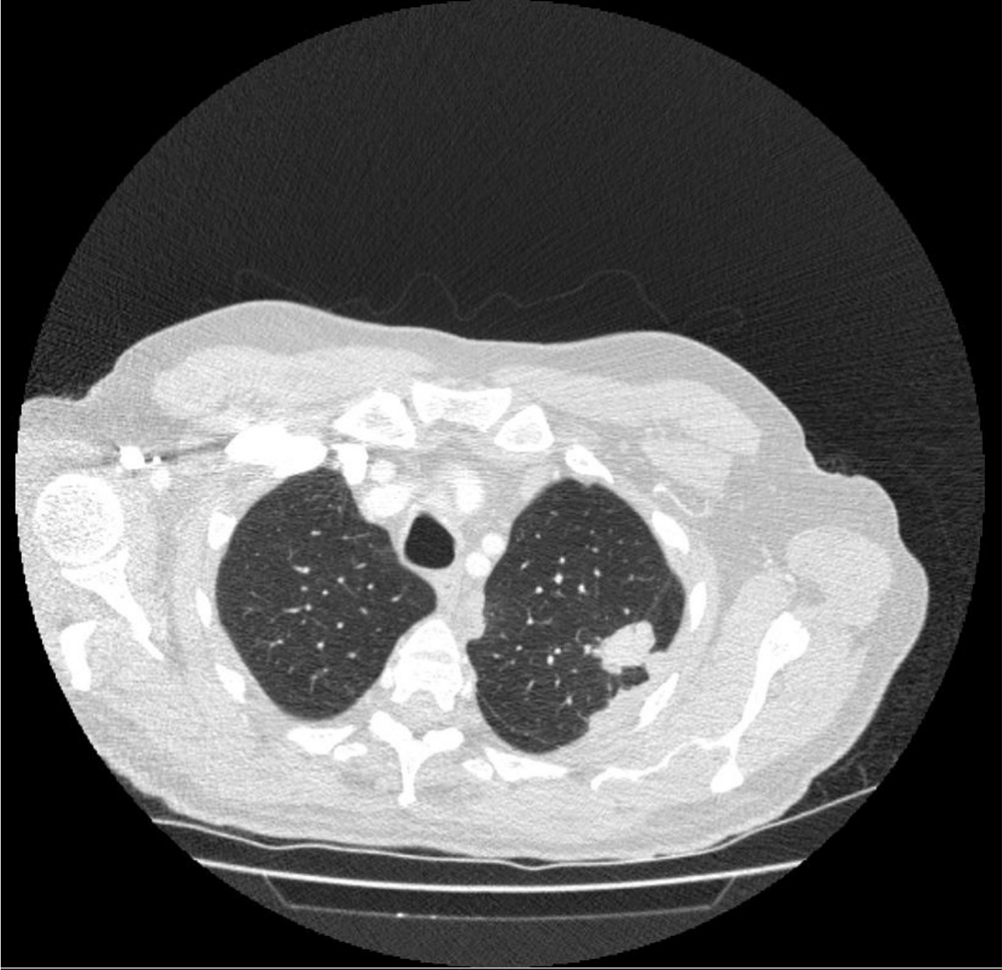
*T*_1 _weighted axial MRI section showing invasion of vertebral body from the mass causing compression of the spinal cord at T5 level.

**Figure 2.**
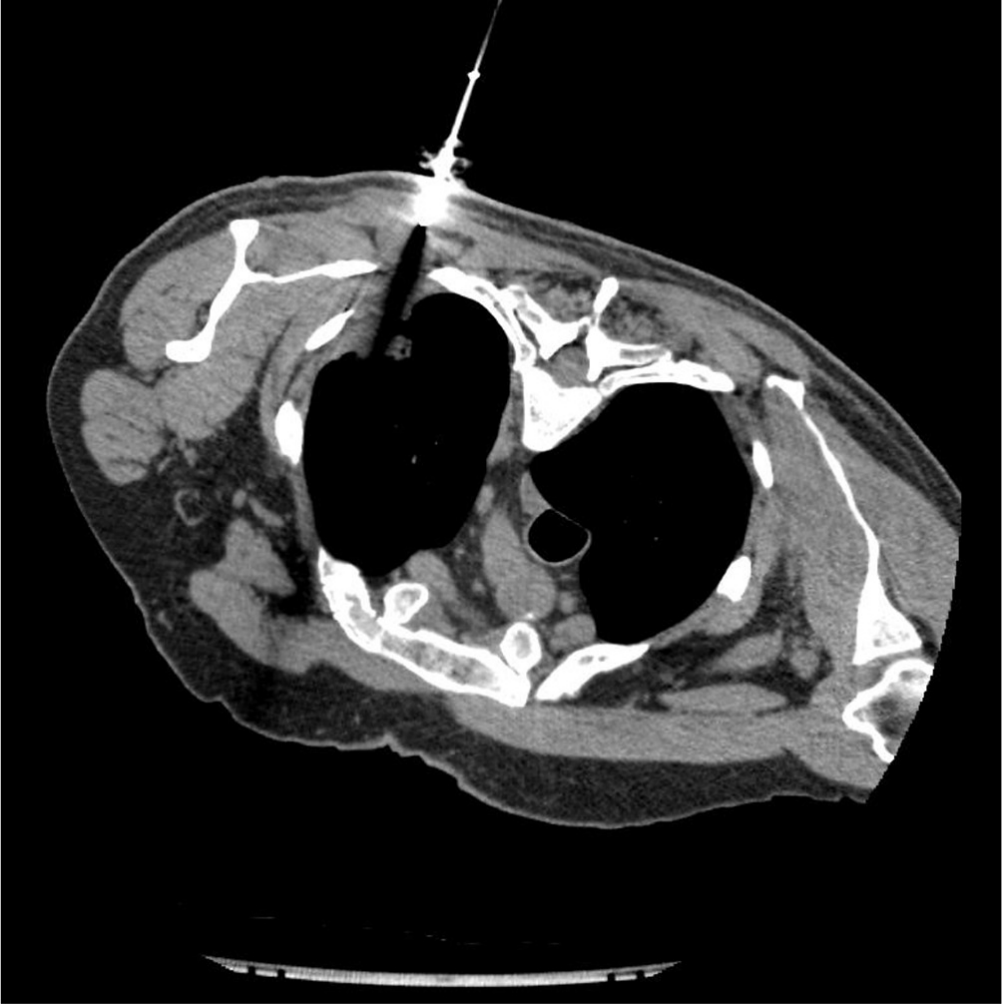
*T*_1 _weighted sagittal MRI depicting the large mass infiltrating the thoracic vertebral bodies (T4/5/6).

**Figure 3.**
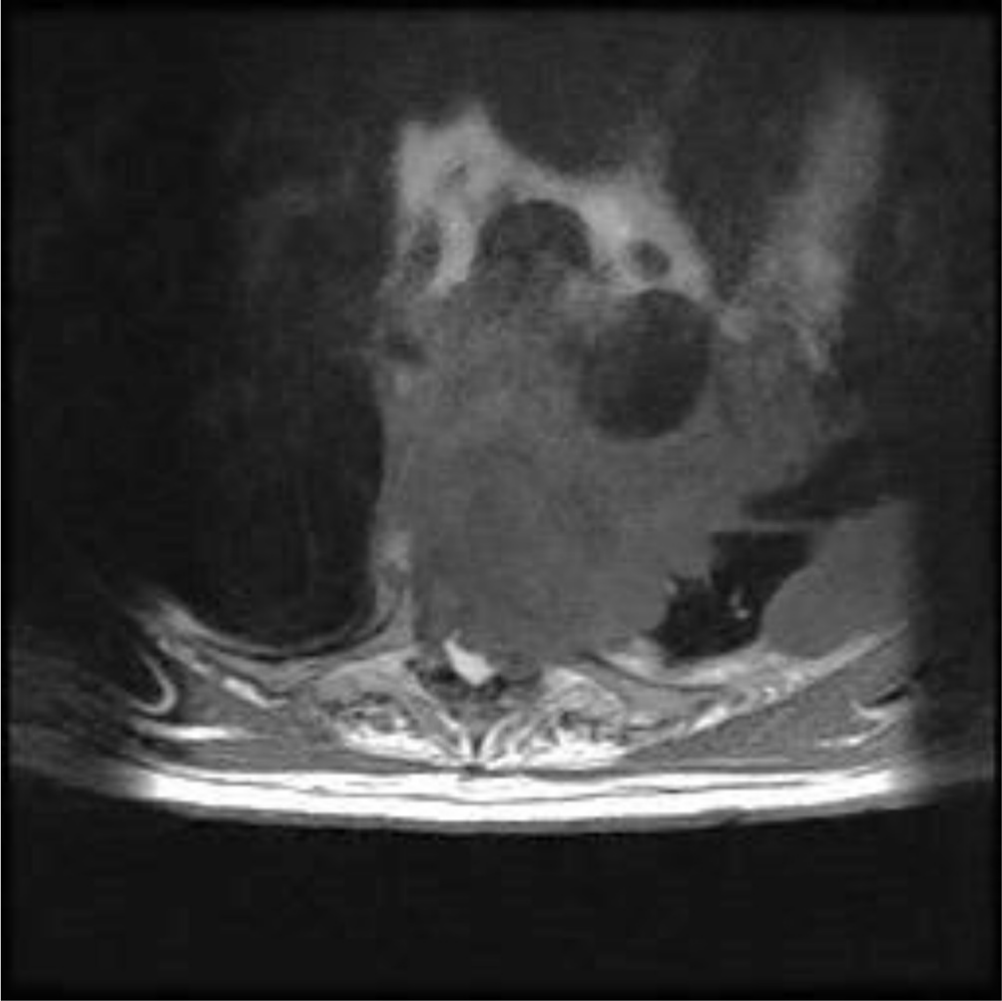
Axial slice of a CT scan showing left-sided lobulated pleural thickening extending into the lung as a 3-cm lobulated mass.

**Figure 4.**
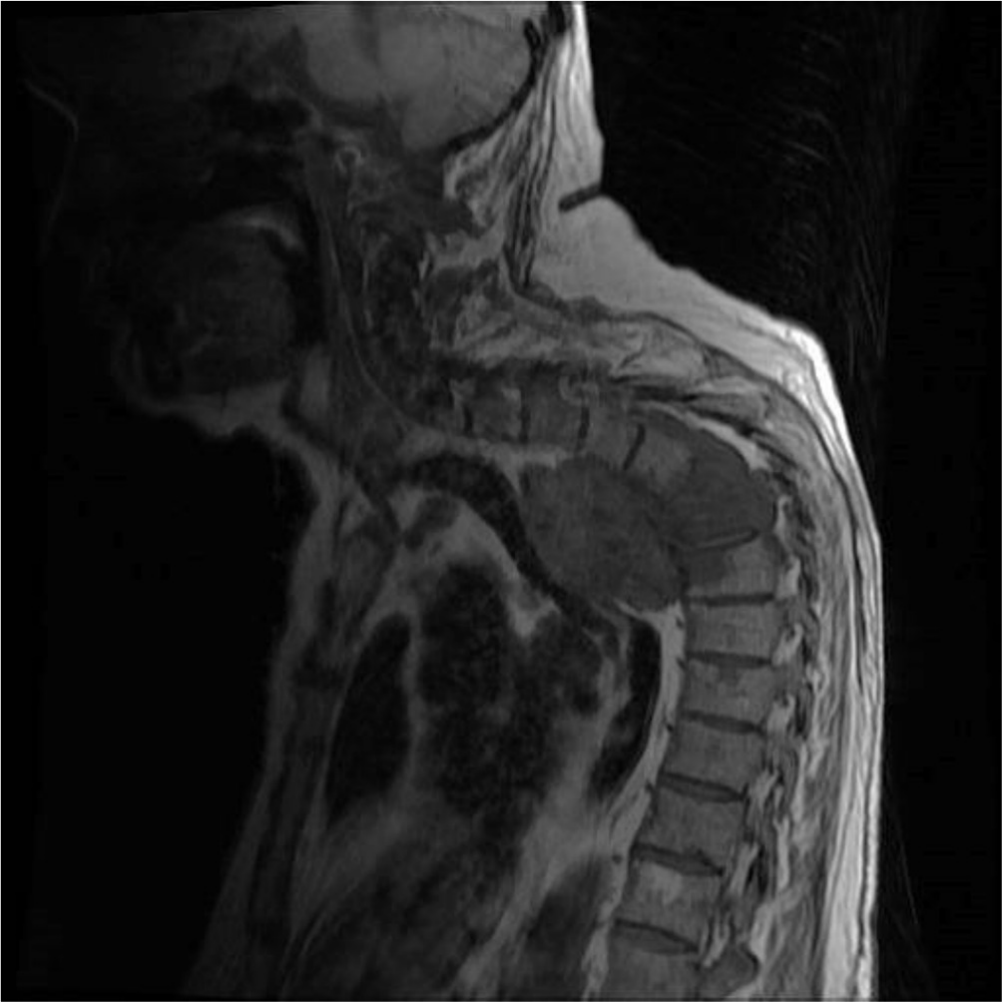
Axial slice of a CT-guided biopsy of a pleural lesion through the posterior thoracic wall.

MRI demonstrated an extension of a pleural mass measuring 6.7 cm by 5.5 cm (Figure 1). The mass had infiltrated the vertebral bodies causing complete collapse at T4/T5 level with intraspinal extensions. Severe narrowing of the spinal canal could be seen at this level, clearly depicted in the sagittal section (Figure 2). Spinal cord compression as a result of invasive mesothelioma was considered the most likely differential diagnosis. Correlating to this case, a differential diagnosis may include prostatic pleural metastases. However, this is unlikely as his disease went into remission in 2006. He had not shown any previous metastatic disease and the prostate-specific antigen level had not risen. When compared with previous CT images the mass was clearly an extension of the previous biopsied mass diagnosed as mesothelioma (Figures 3 and 4).

## Treatment and prognosis

The patient was treated with dexamethasone and offered one cycle of radiotherapy. Initial symptomatic improvement was seen and he was able to stand with aid 4–5 days after starting dexamethasone. His predicted prognosis was less than 3 months. Despite this early improvement he declined over the following week, becoming more fatigued and cachectic. By this time, he had developed worsening symptoms of breathlessness and neurological impairment over the following week. His pain worsened significantly and became difficult to control. Unfortunately, he died prior to receiving palliative radiotherapy to the spinal cord, roughly 4 weeks from the time of admission.

## Discussion

Malignant mesothelioma is an aggressive tumour that develops at serosal surfaces, which include the pleura, peritoneum and tunica vaginalis of the testis.^1^ It usually results from prolonged exposure to asbestos with a latency period of roughly 40 years between fibre exposure and disease presentation.^2,3^ Progression usually tends to be by direct invasion to nearby regions such as the lung parenchyma, the chest wall, the mediastinum and the diaphragm.^1,2^ Involvement of the nervous system is rare. When it occurs it is usually mediated via haematogenous spread, although there has been some reported cases of direct infiltration of the spinal cord through the intervertebral foramina.^2,4,5^

There are four main histological subtypes: epithelioid, sarcomatoid, biphasic and desmoplastic. The most common histological type is epithelioid and is associated with the best prognosis. The sarcomatoid subtype is characterized as spindle morphology and is associated with the worst prognosis.^6^ Hence, mortality in the UK was expected to peak at 2450 deaths per year by 2015.^7^

In the later stages of the disease and with spinal cord involvement, symptoms usually include spinal pain and paraesthesia, weakness and bowel and bladder dysfunction. On radiological imaging, spinal cord involvement can manifest either as a heterogeneous enhancing mass extending through the intervertebral foramen into the spinal canal or as vertebral body collapse, or both.^2,8–10^

There are a few treatment options and these include surgery, radiation therapy and chemotherapy. Those with early epithelioid disease without radiological evidence of lymph node involvement are the best candidates.^11^ Pemetrexed and cisplatin combination chemotherapy remains the most recent and sole approved therapy in the last 13 years.^12^ Those receiving cisplatin and pemetrexed had a significantly longer median overall survival (12.1 versus 9.3 months), longer time to progression (5.7 versus 3.9 months) and greater objective response rate (41 versus 17%).^13^ As our understanding of the immune process in mesothelioma has developed so has the emergence of potential new therapies. Most notably bevacizumab (a receptor-targeting antibody) in combination with chemotherapy has been shown to prolong survival in malignant mesothelioma patients.^13^ Radiation therapy alone is generally used for palliation. There is no evidence to suggest that palliative radiotherapy improves survival, but is shown to be effective in 50% of patients suffering with pain.^11^ In an attempt to reduce both local recurrence and distant metastases, trimodality therapy involving chemotherapy, adjuvant radiotherapy and biological agentshas been implemented in some centres.^13^

Common sites of metastases include lymphatics, lung, liver, adrenal glands and kidneys.^14^ Mesothelioma rarely metastasizes to bone or the nervous system. In a post-mortem study the incidence of bone metastases in patients with mesothelioma was reported at 13.8%.^15^ To date, there are 10 reported cases of cord compression secondary to mesothelioma causing direct invasion, bony metastases or central nervous system metastases.^1,2,5,8,16,17^

We conclude that we need to rule out spinal involvement if a patient with pleural malignant mesothelioma presents with worsening neurological symptoms and likely MRI findings report an abnormal lesion in the thoracic spinal cord.

## Learning points

1. Spinal cord compression is a rare complication of mesothelioma. This has three mechanisms of action: vertebral bone metastases with collapse, central nervous system metastases and direct invasion into the spinal canal.
2. Treatment should be individualized, but options are limited. Appropriate discussion needs to be addressed to give realistic expectation and prepare the patient when early signs of malignancy are identified. Early identification of clinical signs of neurological involvement will allow palliative intervention. Central nervous system involvement presents late and prognosis declines from months to weeks.
3. Progression by invasion is more common than metastases in mesothelioma.
4. Mesothelioma can progress rapidly through direct extension and early radiotherapy may provide better symptomatic control. Hence, there should be a degree of urgency to perform these interventions.
5. Mesothelioma presents late, and can be asymptomatic until significant progression.

## Consent

Written informed consent for the case to be published (including images, case history and data) was obtained from the patient(s) for publication of this case report, including accompanying images.
